# Adolescents perception of the COVID-19 pandemic restrictions and associated mental health and well-being: gender, age and socioeconomic differences in 22 countries

**DOI:** 10.1186/s13034-024-00779-z

**Published:** 2024-07-18

**Authors:** Franziska Reiss, Alina Cosma, Michela Bersia, Michael Erhart, Paola Dalmasso, Janine Devine, Sabina Hulbert, Carolina Catunda, Inese Gobina, Ariela Giladi, Helena Jeriček Klanšček, Ulrike Ravens-Sieberer

**Affiliations:** 1 Department of Child and Adolescent Psychiatry, Psychotherapy, and Psychosomatics, University Medical Center Hamburg-Eppendorf, Hamburg, Germany; 2 Department of Sociology, Trinity College Dublin, Dublin, Ireland; 3 School of Psychology, Trinity College Dublin, Dublin, Ireland; 4 Department of Public Health and Pediatrics, University of Torino, Turin, Italy; 5 Alice-Salomon University, Berlin, Germany; 6 Centre for Health Services Studies, University of Kent, Canterbury, UK; 7 Department of Social Sciences, University of Luxembourg, Esch-Sur-Alzette, Luxembourg; 8 Department of Public Health and Epidemiology, Riga Stradiņš University, Riga, Latvia; 9 Education and Research Unit, Childrens’ Clinical University Hospital, Riga, Latvia; 10 Faculty of Education, Bar Ilan University, Ramat Gan, Israel; 11 Department of Education, Ariel University, Ariel, Israel; 12 National Institute of Public Health, Ljubljana, Slovenia

**Keywords:** HBSC, Well-being, Multiple health complaints, Life satisfaction, Loneliness, COVID-19 measures, Mental health

## Abstract

**Background:**

The COVID-19-pandemic has had a profound impact on the lives of adolescents worldwide. This study examined the subjective perception of the COVID-19 pandemic measures and its association with mental health and well-being (i.e., loneliness, life satisfaction and multiple health complaints) among 13- and 15-years-old adolescents from 22 countries.

**Methods:**

Data from the cross-national Health Behaviour in School-aged Children (HBSC) 2021/22 study were used from representative samples of 22 countries (N = 67,544; 51.9% girls). The self-perceived impact of COVID-19 measure comprised 10 items asking about the impact on several dimensions of adolescent lives (e.g., relationships with family and friends, health, or eating behaviours). Measures of loneliness, multiple health complaints, and life satisfaction were included as indicators of mental health and well-being. A non-parametric multilevel latent class analysis considering individual and country-levels was conducted to identify classes of self-perceived impact of the COVID-19 measures. Multilevel logistic regression models adjusted by age and socioeconomic status were applied to assess the association between COVID-19 measure impact classes and mental health.

**Results:**

Three classes were identified on individual level encompassing a neutral (51%), positive (31%), or negative (18%) perception of COVID-19 measures. A third of the adolescents reported a positive impact of the pandemic measures. The distribution of classes was heterogeneous within and across countries. Within the positive COVID-19 measure impact class, social relationships were the most important dimension, whereas mental health problems were mostly represented within the negative COVID-19 measure impact class. Girls with a negative perception of pandemic measures showed higher levels of loneliness and multiple health complaints and lower life satisfaction. 15-year-old adolescents and those with a low socioeconomic status reported higher levels of loneliness and lower life satisfaction.

**Conclusions:**

The majority of adolescents perceived the pandemic measures as neutral or positive. Girls, 15-year-old adolescents, and those with low socioeconomic status were at higher risk of suffering from pandemic measures and associated problems of loneliness, multiple health complaints, and low life satisfaction. We conclude that adolescent’s mental health and well-being should be considered in the decision-making process by ensuring that the unique challenges of adolescents are adequately addressed in policies.

**Supplementary Information:**

The online version contains supplementary material available at 10.1186/s13034-024-00779-z.

## Background

The coronavirus (COVID-19) pandemic and the consequent implemented confinement measures have caused significant disruptions in daily lives and societal norms, with both immediate and long-term implications for mental health and well-being [1]. Adolescents, in particular, have been at heightened risk of experiencing the psychological effects of the pandemic [2]. Broader social lockdown measures have severely altered their daily routines. Notably, both short- and long-term school closures have led to substantially less social contact, peer support, and loss of physical activity [3, 4]. Numerous reviews and meta-analyses indicate an increase in mental health problems among children and adolescents during the pandemic [1, 2, 5–8]. Although individual studies present heterogeneous results, most of them demonstrate a rise in the prevalence of mental health problems during the initial year of the pandemic, particularly an increase in adolescent anxiety and depression [9]. However, we do not know much about how adolescents actually experienced the impact of pandemic measures and restrictions in different areas of their lives and knowledge is not that advanced on how the experience of confinement measures is related to adolescents’ mental health and well-being [10–13]. So far, some national studies have explored the impact of school closures [4] and quarantine isolation [14] reporting an association with mental health impairments during the implementation of pandemic measures. A recent study by Sanchez-Lopez found a positive relationship between the perceived hardness of confinement and mental health, and a positive association between family relationships, pleasant activities and mental health [15]. The International Health Behaviour in School-aged Children (HBSC), a WHO collaborative study, asks about the perceived impact of the COVID-19 measures and showed that 30% of adolescents reported that the COVID-19 pandemic had had a negative impact on their mental health and well-being, relatively more adolescents (38%) experienced no impact, and 32% reported positive impacts. Those adolescents who perceived a negative impact of the COVID-19 pandemic were more likely to report lower levels of life satisfaction and higher levels of psychological symptoms [16].

Other studies in which adolescents themselves report about the perceived impact of the COVID-19 pandemic restrictionson on their daily lives mainly used a qualitative approach [17, 18] while quantitative studies are still limited. For instance, a study from the UK found that disruptions of friendships were perceived as difficult, yet new forms of maintaining friendships evolved, e.g. via technology [17]. A study conducted in Canada showed that digital interactions could not replace face-to-face interactions and that schools are an essential place for socialization, which cannot be fulfilled via online schooling [18], while a study from Italy showed that quarantine experience itself was associated with 43% feeling less secure, more tense and sadder, 60% were ruminating and 56% had difficulties sleeping among adolescents [19]. Moreover, recent international reports highlighted important social disparities of adolescents’ perceived impact of the COVID-19 pandemic measures [20, 21]. Girls, older adolescents, and those from families with low socioeconomic status perceived a negative impact of the COVID-19 pandemic restrictions in more areas of their lives than their peers (respectively boys, younger adolescents, and adolescents from high socioeconomic status) [21]. Nonetheless, more quantitative studies on the self-perceived impact of the COVID-19 pandemic measures and mental health need to address a more balanced perspective considering simultaneously the negative and positive effects of the pandemic. Last but not least, there is hardly any research on adolescents’ self-perceived impact of the COVID-19 measures across different countries [22] whereas studies on mental health in adolescents use different methodologies, underscoring the necessity for a more standardized approach in this research field [2, 23].

In conclusion, although considerable research has explored the general impact of the COVID-19 pandemic on adolescent mental health, there is still limited systematic, comparable, quantitative evidence from comparable cross-national studies on the self-perceived impact of COVID-19 measures and restrictions on adolescents’ mental health. This is of great importance as adolescents may be more vulnerable and prone to experience negative feelings and fears during a global pandemic that threatens their physical, emotional, and financial lives [24, 25]. Therefore, we focus on the subjective perception of the COVID-19 pandemic restrictions and its interrelation with mental health and well-being. The World Health Organisation defines mental health as “a state of mental well-being that enables people to cope with the stresses of life, realize their abilities, learn well and work well, and contribute to their community” [26]. Building on this conceptualisation, the present study focuses on specific positive (e.g., life satisfaction) as well as negative (e.g., loneliness, multiple health complaints) aspects of mental health. Multiple health complaints together with life satisfaction have been used as measures capturing adolescents’ physical and mental health and well-being for decades [27, 28]. Loneliness has been recognized as an important determinant of mental health and is a major source of psychological stress associated with depression and anxiety [29].

### This study

This study aimed to investigate latent classes of the self-perceived impact of COVID-19 measures and restrictions among representative national samples of adolescents across 22 countries and test the associations between these classes and mental health. In addition, we explored age, gender, and socioeconomic inequalities associated with the aforementioned dimensions. Therefore, the overall research aims are (i) to explore classes of self-perceived impact of the COVID-19 pandemic measures on adolescents’ lives (ii) to explore gender, age, and socioeconomic differences in the impact classes of COVID-19 measures; (iii) to test associations between these classes and different aspects of mental health (i.e., loneliness, life satisfaction and multiple health complaints).

## Methods

### Study design and sample description

The Health Behaviour in School-aged Children (HBSC) study is a large cross-national survey carried out in schools every four years since 1982, in collaboration with the World Health Organization Regional Office for Europe, that monitors adolescents’ self-reported health behaviours, health outcomes, and social environments. Data is collected through a self-administered questionnaire using a standard methodology detailed in the HBSC international study protocol [30]. All countries provide nationally representative samples of children and adolescents aged 11-, 13- and 15- years-old with schools/classes being the primary sampling unit [30]. The most recent survey (2021/2022) included an optional set of questions that evaluated the self-perceived impact of the COVID-19 pandemic measures on adolescent lives plus measures of loneliness, life satisfaction and multiple health complaints, which was implemented by 22 countries. This study focuses on adolescents aged 13- and 15- years and their perception of the COVID-19 measures and restrictions (N = 67,544; 51.9% girls) in 22 countries geographically distributed across Europe and Central Asia, from Spain, Luxembourg and Finland to Kazakhstan.. Data collection started in November 2021 at the earliest (Cyprus, Estonia and Sweden) and ended in November 2022 (Germany, Ireland and Norway) or December 2022 (Greece) at the latest. Data collection periods for each country are outlined in the Supplementary Material (Figure S1) as well as individual country level sample sizes (Figure S2).

### Measures

All of the measurements used in the international HBSC study, and here, have been standardised.

**Table 1:** Sociodemographic characteristics and mental health levels of the sample (N = 67,544)

	Overall	Boys	Girls	Missing	p
	N = 67,544	n = 32,444	n = 35,100	N = 67,544	
	in %	in %	in %	in %	
Gender				0.0	
Boys	48.1				
Girls	51.9				
Age				0.0	
13 years	46.8	47.8	46.0		0.301
15 years	53.2	52.2	54.0		
Socioeconomic status				2.0	
Low	24.9	23.7	26.0		0.001
Medium	62.5	61.9	63.1		
High	12.6	14.4	10.9		
Loneliness				0.7	
≤ Sometimes	84.5	90.6	78.7		< 0.001
≥ Most of time	15.5	9.4	21.3		
Multiple health complaints				1.2	
< 2 symptoms more than weekly	53.0	69.8	37.5		< 0.001
≥ 2 symptoms more than weekly	47.0	30.2	62.5		
High life satisfaction				0.8	
≤ 8	85.0	83.1	86.8		< 0.001
≥ 9	15.0	16.9	13.2		

#### Self-perceived impact of the COVID-19 pandemic measures

The *COVID-19 Impact Scale* is a self-reported measure developed to assess the impact of COVID-19 measures experiences on a range of domains relevant in the life of adolescents (see Supplementary Material, Table S1). It was developed by the HBSC network and it uses a five points Likert-like response format ranging from “very negative” (1) to “very positive” (5) with neutral (i.e., 3—“neither positive nor negative”) as mid category to capture the direction and intensity of respondents’ opinions of the impact of COVID-19 on life in general, overall health and mental health, relationships with family and friends, school performance, physical activity, eating behaviours, future expectations and family finances [21].The scale consists of 10 items, showing an excellent internal consistency in our sample (α = 0.91).

#### Mental health

*Loneliness* was measured by a single item asking about the perceived general loneliness in the last 12 months. The measurement has been adopted from the Global Student Health Survey (GSHS). GSHS-based studies have shown the construct validity of the item [31]. Multiple-item measures of loneliness have similar validity and reliability as a single-item measure [32–34]. In line with previous recommendations, a categorical measure was created on the basis of reporting loneliness “most of the time” or “always” vs. all the others (“sometimes”, “rarely”, “never”) [30].

*Multiple Health Complaints (MHC)* were measured by an eight-item instrument that captures the frequency of the physical and psychological complaints in the past six months (i.e., headache, abdominal pain, backache, dizziness, feeling low, irritability or bad mood, feeling nervous, and difficulties in getting to sleep). Adolescents rated the frequency of each health complaint on a 5-point scale from “about every day“(1) to “never” (5). The multiple health complaints measure has been shown to have acceptable test–retest reliability, internal consistency, and a unidimensional model is supported in most countries [35]. Furthermore, a good internal consistency was found in our sample (α = 0.85). Based on previous recommendations, data were recorded into a binary categorical measure comparing adolescents presenting at least two health complaints more than once a week vs. less [30].

*Life satisfaction (LS)* was assessed with the one-item visual-analogue Cantril ladder scale with wording suitable for children as young as 11 years old [36]. Respondents ticked the number next to the step that best describes the position on the ladder where they feel they stand at the moment “worst possible life for you” (0) and “best possible life” (10). The measure has extensive evidence of validity and reliability [37, 38]. In line with previous recommendations, the responses were recorded so that they reflect high life satisfaction (i.e., a score of at least 9) vs. all others [27].

#### Sociodemographics

Adolescents age was assessed by asking them to indicate the year and month they were born, and gender was assessed by asking them whether they are boy or a girl.

*Socioeconomic status (SES)* was measured by the *Family Affluence Scale III* (FAS), which is a set of six items designed and validated within the HBSC study [39]. The scale is suitable for use with children in the 11–15 years old range and measures participants’ socioeconomic status by monitoring self-reported access to family resources available in the home (i.e., car, own bedroom, computers, bathrooms, dishwasher, and holidays). An ordinal cumulative score can be computed by adding individual items or a continuous Ridit score can be derived via the ranking of cumulative proportions. Previous validation work supports the construct and concurrent validity of the measure [40, 41].

## Data analysis

### Descriptive statistics

Prevalence of sociodemographic characteristics (age, SES) and mental outcomes (i.e., loneliness, multiple health complaints, life satisfaction) were assessed by gender, and potential differences were evaluated through chi-square tests, considering survey design effects (including stratification, clustering, and weighting).

### Latent class analysis

**Fig. 1:**
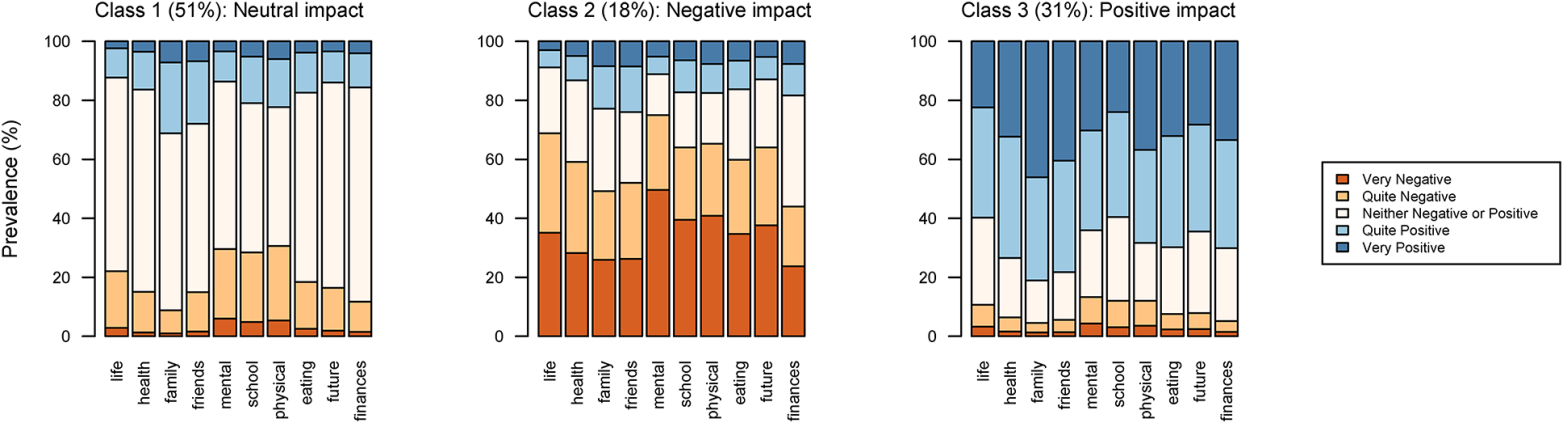
Distribution of the COVID-19 measure impact scale items by latent class

**Fig. 2:**
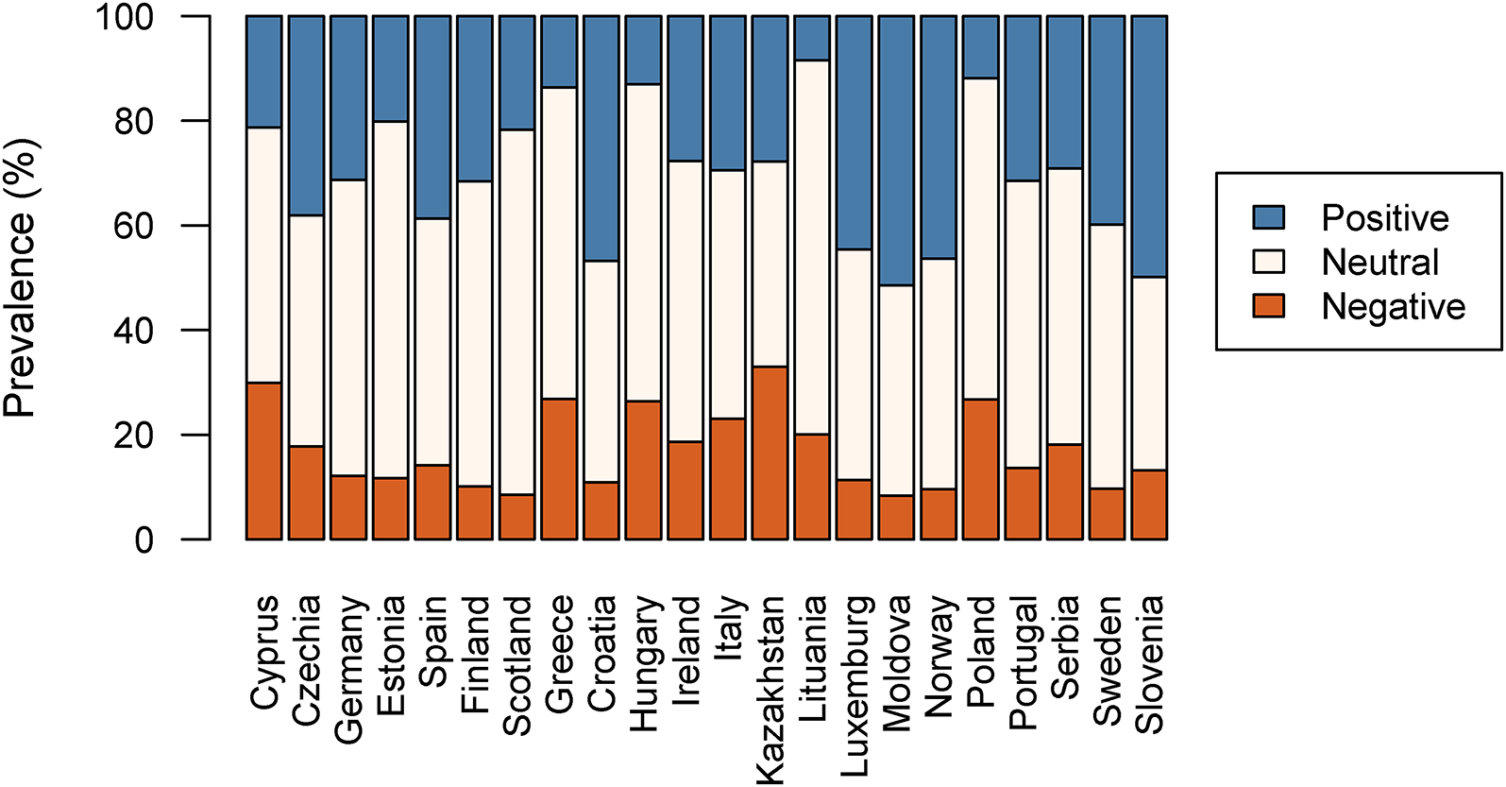
Distribution of the COVID-19 impact classes by country

Non-parametric multilevel latent class analysis (MLCA) was conducted on the ten items of the COVID-19 measure impact scale to identify COVID-19 measure impact classes. Models were run on observations with complete data for all the COVID-19 impact scale items (n = 67,544), entered as five levels of ordinal variables. A random effect ruled by country was introduced in the model. One to five latent class models were compared in terms of information criteria (e.g., AIC and BIC), and entropy to determine the optimal number of latent classes [42, 43] (see Supplementary Material Table S1). The selection of the class solution was made on the basis of statistical criteria assessment in conjunction with interpretability evaluation [43]. It moved towards the three classes solution, showing a quite good fit (AIC = 1 616,330, BIC = 1 617,826, entropy = 0.89). Class membership was determined by the modal probability of belonging to a specific class. Means of modal probability of belonging to the assigned class 1, 2, and 3 ranged from 0.93 to 0.96. The previous finding confirmed that for large sample sizes a two-stage model is sufficient to use the latent classes as a predictor in a second-stage regression model. Distribution of the COVID-19 measure impact items by class (Fig. 1) and distribution of impact classes by country (Fig. 2) were then performed at a descriptive level. Distribution of age, gender, and SES by impact class and potential differences were evaluated through chi-square tests, considering survey design effects (Table 2).

### Regression models

Quasibinomial regression models weighted for the study design were performed introducing COVID-19 measure impact class, age, and socioeconomic status as independent variables and, alternatively, the three explored mental outcomes (i.e., loneliness, life satisfaction, multiple health complaints) as dependent indicators. Further, based on the previous models, the mean predicted probability of the three outcomes was computed in the three COVID-19 measure impact classes. Two-tailed tests were performed, and a 5% significance level was set. With reference to the given the systematic evidence around gender differences in adolescent well-being [16, 44], all inferential analysis has been stratified by gender.

### Sensitivity analysis

First, a sensitivity analysis was performed for the MLCA on the basis of 9 items removing the impact on mental health and comparing the results with the MLCA on the basis of 10 items including the mental health item. For both, regression models were performed. Second, with reference to Research Question 3, a sensitivity analysis was conducted performing regression models for other well-being indicators. More specifically, given that the 2021/22 HBSC survey included other mental health and well-being outcomes, we ran a series of additional models with perceived health as excellent, WHO-5 Well-being Index Score and Self-confidence as outcomes.

All analyses were performed using R (version 4.2.1).

## Results

### Descriptive statistics

Overall, 67,544 adolescents from 22 countries and regions aged 13–15 (53.2% 15-years-olds) were included in the present study. Due to statistical inclusion criteria, 9,314 observations with incomplete COVID-19 Impact Scale data were removed, accounting for 12,1% of the overall observations with available age and gender information (N = 76,858), obtaining a final sample size of 67,544 adolescents, on which analysis were performed. Compared to those with complete COVID-19 Impact Scale data, observations with incomplete COVID-19 data are more likely to be boys (54% vs. 48%) and 13 years olds (58% vs. 49%), while similar distributions were found for low socioeconomic status (26% vs. 27%), loneliness (18% vs. 19%), high life satisfaction (31% vs. 28%), and multiple health complaints (47% vs. 49%).

Sociodemographic characteristics and mental health variables (i.e., loneliness, multiple health complaints, and life satisfaction) were summarised in Table 1. Gender and age groups were equally distributed in the sample, and more than half of the sample could be categorized as medium socioeconomic status (62.5%). Further information on sample size per country, age and gender is presented in the Supplementary Material (Table S2).

Overall, about 1 out of 6 adolescents indicated that they felt lonely most of the time or always, almost 1 out of 2 adolescents presented multiple health complaints, and about 1 out of 6 adolescents showed high life satisfaction. Girls reported significantly worse mental health outcomes than boys, accounting for a higher prevalence of loneliness, more multiple health complaints, and lower prevalence of very high life satisfaction.

### Self-perceived impact of COVID-19 measures

The first research aim was to explore classes of perceived impact of the COVID-19 pandemic measures on adolescents’ lives. Following the non-parametric multilevel latent class analysis, a three-class solution was adopted, showing the best fit and interpretability among the latent class models and a very high modal probability for the 3-class solution (Fig. 1 and Supplementary Material Table S3). Of the total sample, 51% of participants were assigned to class 1 (neutral impact of COVID-19 measures), 18% were assigned to class 2 (negative impact of COVID-19 measures), and 31% were assigned to class 3 (positive impact of COVID-19 measures). Adolescents in class 1 (neutral impact) presented very high rates of “neither positive nor negative” responses to the COVID-19 impact scale items (from 47.1% to 72.6%). In contrast, class 2 (negative impact) and 3 (positive impact), respectively, presented high levels of “quite negative” or “very negative” and “quite positive” or “very positive” answers on the COVID-19 impact scale. Thus, based on the prevalence of such items’ answers, the three classes were interpreted as neutral, negative, and positive COVID-19 measure impact class.

In the positive COVID-19 measure impact class (class 3), the items that asked about the impact of the pandemic measures on friends’ and family relationships were answered most often positively (> 80%), i.e., adolescents, who reported a positive impact of the pandemic measures, found the most positive impact in respect to their families and friends. Furthermore, in the negative COVID-19 measure impact class (class 2), the item which referred to the impact of the pandemic measures on mental health received the highest rates of negative answers (> 75%), as well as the impact of measures on school performance and physical activity. In the neutral COVID-19 impact class (class 1), the largest number of “neither positive nor negative” responses were registered for the impact of COVID measures on future expectations and family finances. The distribution of the single COVID-19 measure impact items by COVID-19 measure impact class are presented in Supplementary Material (Table S4).

The distribution of the impact classes by country depicted a heterogeneous scenario (Fig. 2 and Supplementary Material Table S5). With respect to single countries, Kazakhstan (33.0%) was the country with the highest negative impact class rate, followed by Cyprus (29.9%), Greece (26.8%), Poland (26.7%), and Hungary (26.4%), while Moldova had the lowest negative impact rate (8.3%). Regarding positive impact of the COVID measures, the highest positive impact rate was registered for Moldova (51.5%), Slovenia (49.9%), and Croatia (46.8%), while Lithuania registered the lowest positive impact (8.4%).

To address our second research aim, age, gender, and socioeconomic distribution by COVID-19 measure impact class were calculated (see Supplementary Material Table S6). Girls were assigned more frequently to the negative COVID-19 measure impact class than boys (61.0% vs. 39.0%). The 15-year-olds were assigned more frequently to the negative COVID-19 measure impact class compared to the 13-year-olds (57.5% vs. 42.5%), and those with a low SES were assigned more frequently to the negative COVID-19 measure impact class compared to those with higher SES (31.1% vs. 10.5%). Conversely, the latter were located mainly in the positive COVID-19 measure impact class.

### Association between the self-perceived impact of the COVID-19 measures and mental health outcomes

**Fig. 3:**
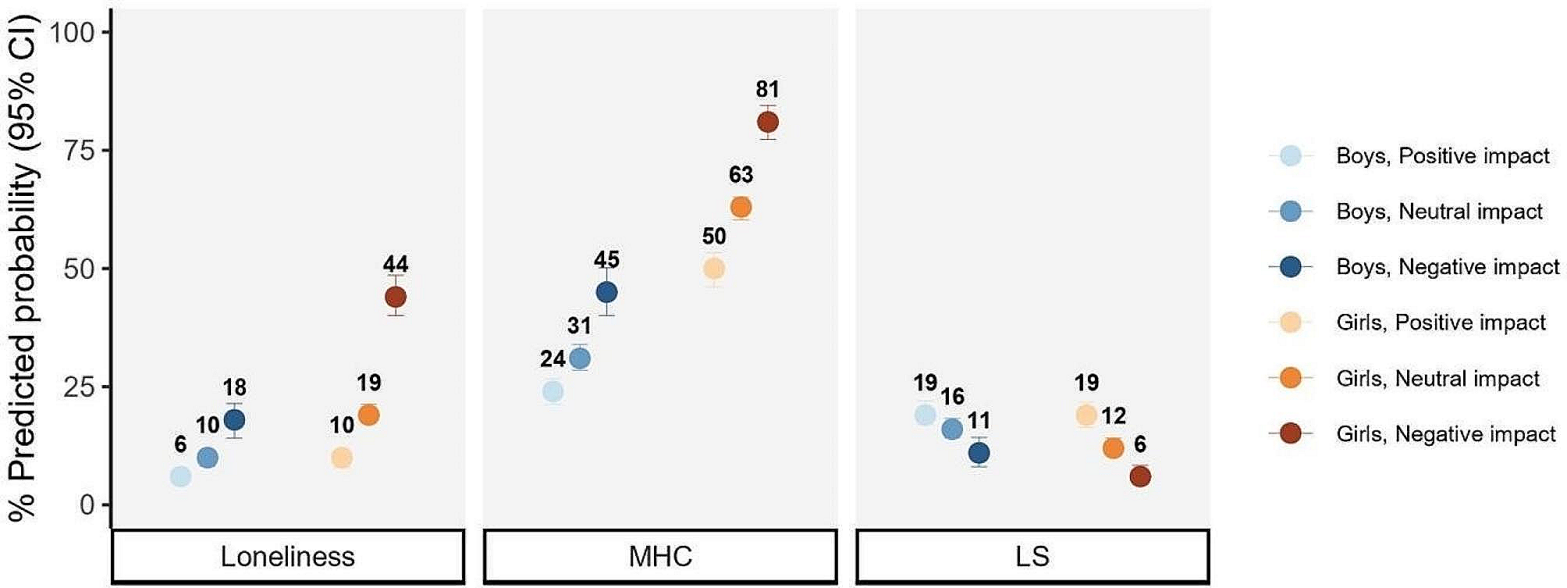
Percentage predicted probability (95% CI) of mental outcome by impact class among boys and girls. *MHC* multiple health complaints, *LS* life satisfaction. Results adjusted by age and socioeconomic status

To address the third research aim, the associations between COVID-19 measures impact classes and adolescent mental health were tested. Those in the negative self-perceived COVID-19 measure impact class showed a statistically significant poorer mental health reflected in higher levels of loneliness and multiple health complaints as well as lower levels of life satisfaction (Table 2 and Fig. 3, Supplementary Material Table S7). In contrast, those in the positive COVID-19 measure impact class reported more favourable outcomes. The association between COVID-19 impact and mental health outcomes showed stronger effects for girls compared to boys.

**Table 2:** Association between COVID-19 measure impact classes and mental health outcomes by gender (N = 67,544)

	Boys		Girls	
	OR	95% CI	OR	95% CI
Loneliness				
Negative impact (vs. neutral)	**2.01**	1.49−2.71	**3.35**	2.74−4.10
Positive impact (vs. neutral)	**0.57**	0.43−0.76	**0.45**	0.35−0.57
Medium SES (vs. low)	**0.69**	0.53−0.90	**0.63**	0.52−0.77
High SES (vs. low)	**0.58**	0.38−0.88	**0.68**	0.50−0.93
15-year-oldsolds (vs. 13-year-olds)	**1.61**	1.22−2.12	1.03	0.85−1.25
MHC (≥ 2 at least twice a week)				
Negative impact (vs. neutral)	**1.81**	1.41−2.32	**2.53**	2.00−3.21
Positive impact (vs. neutral)	**0.69**	0.58−0.84	**0.59**	0.49−0.70
Medium SES (vs. low)	0.95	0.77−1.16	0.85	0.71−1.02
High SES (vs. low)	0.91	0.69−1.21	1.06	0.82−1.38
15 yrs olds (vs. 13)	**1.24**	1.03−1.48	**1.30**	1.10−1.53
Life satisfaction (≥ 9)				
Negative impact (vs. neutral)	**0.65**	0.46−0.91	**0.48**	0.33−0.69
Positive impact (vs. neutral)	**1.25**	1.00−1.56	**1.66**	1.34−2.05
Medium SES (vs. low)	**1.39**	1.08−1.79	1.08	0.84−1.38
High SES (vs. low)	**1.81**	1.31−2.48	**1.72**	1.25−2.35
15 yrs olds (vs. 13)	**0.75**	0.61−0.93	**0.70**	0.56−0.89

*SES* socioeconomic status, *MHC* multiple health complaints, *yrs* years, *OR* odds ratio

In bold p value < 0.05

The strongest effect of perceived impacts of the COVID-19 measures on adolescent mental health was found for loneliness. More specifically, for both boys and girls, those in the negative impact class had higher odds of reporting loneliness, however this effect was stronger for girls than for boys. Conversely, those included in the positive COVID-19 measure impact class had the lowest odds of reporting loneliness, i.e., only half as often as girls from the neutral COVID-19 measure impact class. Furthermore, the mean weighted probabilities of feeling lonely varied by the perceived impact of the COVID-19 pandemic measures, ranging from 9.7% (positive impact) to 44.3% (negative impact) among girls and from 5.8% (positive impact) to 17.8% (negative impact) among boys.

A strong effect of perceived impacts of the COVID-19 measures on adolescent mental health was also found for multiple health complaints. More specifically, when comparing the negative COVID-19 measure impact class to the neutral COVID-19 measure impact class, girls were more than twice more likely to report multiple health complaints, whereas boys had a smaller risk of reporting those. Conversely, girls in the positive COVID-19 measure impact class reported multiple health complaints only half as often as girls from the neutral COVID-19 measure impact group. In addition, an increasing weighted predicted probability of presenting multiple health complaints was observed among positive, neutral, and negative impacted girls (49.7%, 62.7%, and 80.9%) and boys (24.0%, 31.2%, and 45.1%, respectively). This accounted for an increased odds of 81% and 153% of multiple health complaints among those in the negative impact class, while the odds decreased to 31% and 41% for those in the positive COVID-19 measure impact class compared to neutral COVID-19 measure impact class.

The association between the impact of pandemic measures and life satisfaction showed a smaller effect. Both, boys and girls, in the negative COVID-19 measure impact class reported high life satisfaction half as often as peers from the neutral COVID-19 measure impact class, while the odds to report life satisfaction increased slightly among positively impacted adolescents. The weighted probability of life satisfaction ranged from 19.1% to 6.4% among girls and from 19.5% to 11.2% among boys across the three impact classes.

In all the models (Table 2), the association between mental health outcomes and the perceived impact of COVID-19 measures were strongest for the 15-year-old adolescents compared to the 13-year-olds. Adolescents of both genders, growing up in in high and middle socioeconomic status family had lower odds of reporting loneliness and higher odds of reporting a high life satisfaction than their peers with a low socioeconomic status. Socioeconomic status was not associated with multiple health complaints at a statistically significant level among boys and girls.

The sensitivity analysis performing a MLCA on the basis of 9 items (removing the one on the self-perceived impact of pandemic measures on mental health) and comparing the results with the reported finding above, revealed a concordance of about 96% in the class assignment between the 10 items and 9 items models, and the results of the regression models showed very similar results. In addition, we ran a series of sensitivity analyses to investigate the robustness of our findings in relation to Research Question 3. The results reported in the Supplementary Material (Table S8) indicate similar associations with further HBSC mental health outcomes included in the main manuscript, and therefore providing support for the direction of associations already reported.

## Discussion

Our study explored whether adolescents can be grouped by how they perceived the pandemic measures and how these emerging classes were associated with their mental health. We found three classes of self-perceived impact of pandemic measures on adolescents’ lives, encompassing a neutral (51%) positive (31%) or negative (18%) perception of COVID-19 measures. These results indicate that the majority of adolescents perceived the pandemic measures as either neutral or positive. Across the 22 countries examined, a third of the participants reported that the COVID-19 measures had a positive impact on different areas of their lives. In specific, these results also highlight that relationships with friends and family seem to be one of the most important dimensions for a positive perception of the pandemic measures. Thereby, having perceived a positive impact of the COVID-19 measures might be especially linked to the quality of close relationships. This result is corroborated by systematic reviews that have shown for some families being able to spend more quality time together has been positive [45, 46]. We assume that this positive impact may have counterbalanced the negative impact of the COVID-19 measures on adolescents’ lives, e.g. school-closures. Furthermore, recent research points out that a good family climate and family cohesion are some of the most important resources for children’s and adolescent’s mental well-being. In addition, the scientific literature is abundant in documenting the negative consequences of the COVID-19 pandemic, however, to our knowledge, studies measuring both positive and negative impacts of measures are rare. One recent study by Sanchez-Lopez [15] also measured the positive and negative emotional impacts of the pandemic and found that the affectivity mediates the association of contextual factors (like hardness of confinement) to mental health [15]. Positive affectivity was in that study also associated with relationships at home (and additionally with pleasant activities), while negative emotions were associated with hardness of confinement and worries about contagion [45–47].

In our study, approximately one-fifth of the participants reported that the COVID-19 measures had a negative impact on different areas of their lives. Adolescents with a negative perception of the COVID-19 measures reported most prominently that their mental health, school performance and physical activity were negatively affected. Furthermore, these adolescents tended to show worse mental health as indicated by being lonelier, having more multiple health complaints and lower life satisfaction than peers with a neutral or positive perception of the pandemic measures. It could be discussed whether this negative perception is influenced by poor mental health, i.e., we know that depressed adolescents tend to have more negative cognitions and potentially perceive things distorted. Our findings are in line with international research results pointing out an increase in mental health problems [1], problems with school performance [48] and less physical activity [49, 50] during the pandemic.

We found a quite heterogeneous picture of the distribution of the subjective perception of COVID-19 measures both within and across countries, whereby the assignment to one of the pandemic measure impact classes is not equally distributed by sociodemographic determinants. In specific, we found strong gender, age, and socioeconomic differences between the impact classes of COVID-19 measures across the 22 countries. Especially girls, 15-year-old adolescents, and those with a low socioeconomic status reported more often a negative perception of the COVID-19 measures than boys, 13-year-olds, and those with a high socioeconomic status. Further, our results showed that girls reported poorer mental health indicated by more loneliness, multiple health complaints and lower life satisfaction than boys. This is consistent with previous studies showing that the risk for mental health problems during (and also before) the pandemic was higher for girls than for boys [51–53]. It could be argued that females suffered more from COVID-19 measures. Cross-cultural research found that females were more likely to report emotional and behavioural problems lasting longer than one year and had more COVID-19 anxiety, suggesting poorer mental health than males [54, 55]. There is evidence that girls are more likely than boys to rely on their social networks for support when dealing with significant life stressors [56]. The pandemic constraints (e.g., online schooling, social distancing) affected adolescent females' ability to rely on their social network for emotional support, which could have led to a deterioration in their mental well-being [52]. The findings from the study by Halldorsdottir et al. [57], similar to our study, confirm that girls were more likely than boys to perceive that the pandemic measures had a negative impact on their daily lives.

With regard to age, in our study 15-year olds showed a deterioration in all indicators of mental health indicated by more loneliness, multiple health complaints and lower life satisfaction. These results are congruent with those of previous national and international studies, which also showed that girls and older adolescents more often report rather poor health, multiple psychosomatic complaints, as well as lower life satisfaction [8, 58].

The results on socioeconomic status showed an interesting, differentiated picture: Adolescents with a lower socioeconomic status reported more often from loneliness and a lower life satisfaction. This first result is in line with a study by Jeriček Klanšček & Furman [59], which suggests that self-reported deprivation and economic hardship are significant predictors of poor well-being and the risk for mental health problems. Even before the pandemic, adolescents with a low socioeconomic status were especially vulnerable to worse mental health [60]. Recent studies revealed that increased financial worry during the COVID-19 pandemic was significantly associated with increased child mental health problems [61]. It can be assumed that they and their families have limited resources to deal with fundamental crisis-related measures such as school closures and social distancing, e.g. due to a low educational level of the parents, limited living space, or a high parental burden. However, our study also surprisingly found no significant differences for multiple health complaints within the socioeconomic status groups. This needs to be explored in future studies.

When it comes to international comparisons, we found that, on one hand, some countries presented an intuitive pattern of low levels of positive perception of COVID-19 measures and restrictions off-set by high levels of negative perceptions of COVID-19 measures, like in Hungary and Greece or vice-versa, like in Moldova. However, in some other countries, positive and negative perceptions of COVID-19 measures did not exclude each other and were present either simultaneously in high levels, like in Kazakhstan and Italy or in low levels, like in Lithuania and Estonia. These different patterns could be due to national and cultural specific factors such as COVID-19 cases and deaths or also to variations in the implementation of the COVID-19 restrictions. As analysing mechanisms supporting the heterogeneity of these results was beyond the scope of our paper and future research could better address this gap.

### Strengths and limitations

This research has several strengths and limitations. The main strength of our study is that it uses a direct measure of COVID-19 measure impact, which adds important insights to the current research. Another strength is the inclusion of a large sample of adolescents in 22 countries, which allows for a cross-national comparison when assessing the impact of the COVID-19 pandemic measures on adolescents’ mental health as indicated by loneliness, multiple health complaints, and life satisfaction. Moreover, the HBSC study uses a standardized protocol for data collection across all countries included which facilitates valid cross-national comparisons across countries. Finally, we systematically used sound methodology, i.e., the non-parametric multilevel latent class analysis among others to deepen the understanding of the actual perceived experience of pandemic measures in adolescents and its relation to mental health from a holistic perspective taking into account different sociodemographic aspects.

This study has several limitations. First, it is a cross-sectional design, which does not allow us to obtain evidence on temporal and cause-effect relationships. Another issue concerns the time range of the HBSC data collection and that the pandemic waves and measures varied across countries. This may have impacted the assessment both on individual and country levels. In most of the countries studied, the data were collected when the war in Ukraine began, which may have influenced the well-being of adolescents [62]. When estimating the association between the COVID-19 pandemic and adolescent mental health outcomes, most studies have used non-probability or convenience samples, and different methodologies. However, the unified HBSC methodology and the representative adolescent samples ensure valid cross-country comparisons.

## Conclusions

Our findings provide a unique and comprehensive study of a cross-national comparison examining the self-perceived impact of the COVID-19 pandemic measures on adolescents’ mental well-being in more than twenty countries. Surprisingly, the majority of 13- and 15-year-old adolescents perceived the pandemic measures as either neutral or positive. However, the measures also negatively affected vulnerable adolescents. For example, girls were more likely to have a negative impact of the COVID-19 measures as well as 15-year olds and those with a low socioeconomic status, who seem to have suffered more from the COVID-19 measures than boys, 13-year olds and those with a high socioeconomic status. The self-perceived negative impact of the COVID-19 measures on mental health was associated with the assessed negative mental health outcomes. While we do not know yet, whether the negative impact of the pandemic measures still persists or was short-lived, we would like to encourage future research to replicate our findings. Similarly, there is a need for continuous health monitoring to examine if young people are still burdened after the pandemic. It is a societal responsibility to support those mentally burdened, our vulnerable young people, who still need to recover from the pandemic [63]. In addition, and as a conclusion, our study also showed that adolescents reported that COVID-19 measures impacted their health. Therefore, their (mental) health should be considered in the decision-making process by ensuring that the unique challenges of young people are adequately addressed in policies, thus enhancing inclusive governance and an investment in creating an informed and engaged youth for the future.

### Supplementary Information

Supplementary Material 1.

## Data Availability

The dataset supporting the conclusions of this article is available upon request at the HBSC Data Management Centre from 2024. More information can be found on the webpage: https://hbsc.org/data/.

## Abbreviations

HBSC: Health Behavior in School-Aged Children
MLCA: Multilevel latent class analysis
SES: Socioeconomic status
GSHS: Global Student Heath Survey
MHC: Multiple health complaints
LS: Life satisfaction
FAS: Family Affluence Scale III
