# Supplementary material for: Adolescents perception of the COVID-19 pandemic restrictions and associated mental health and well-being: gender, age and socioeconomic differences in 22 countries
Source: Child Adolesc Psychiatry Ment Health. 2024 Jul 18;18:86. doi: 10.1186/s13034-024-00779-z (PMC11264767; doi:10.1186/s13034-024-00779-z)
Supplement: Supplementary file 1 — Supplementary Material 1. [file 13034_2024_779_MOESM1_ESM.docx]

**Supplementary material**

**Self-perceived impact of COVID-19 measures and adolescent mental health: gender, age and socioeconomic differences in 22 Countries**

**Authors:** Franziska Reiss ^a^*, Alina Cosma ^b,c^*, Michela Bersia^d^, Michael Erhart ^a, e^, Paola Dalmasso^d^, Janine Devine ^a^, Sabina Hulbert ^f^, Carolina Catunda^g^, Inese Gobina^h,i^, Ariela Giladi^k, l^, Helena Jeriček Klanšček^l^, Ulrike Ravens Sieberer ^a^

* Shared first Authorship

^a^ Department of Child and Adolescent Psychiatry, Psychotherapy, and Psychosomatics, University Medical Center Hamburg-Eppendorf, Hamburg, Martinistraße 52, 20246 Hamburg, Germany

^b^ Department of Sociology, Trinity College Dublin, Dublin, Ireland

^c^ School of Psychology, Trinity College Dublin, Dublin, Ireland

^d^ Department of Public Health and Pediatrics, University of Torino, Via Santena 5 bis, 10126 Torino, Italy

^e^ Alice-Salomon University, Alice-Salomon-Platz 5, 12627 Berlin, Germany

^f^ Centre for Health Services Studies, University of Kent, UK

^g^ Department of Social Sciences, University of Luxembourg, Esch-sur-Alzette, Luxembourg

^h^ Department of Public Health and Epidemiology, Riga Stradiņš University, Latvia

^i^ Education and Research Unit, Childrens’ Clinical University Hospital, Riga, Latvia

^k^ Faculty of Education, Bar Ilan University, Ramat Gan, Israel

^l^ Department of Education, Ariel University, Ariel, Israel

^m^ National Institute of Public Health, Trubarjeva 2, 1000 Ljubljana, Slovenia

**Figure S1 Dates on which the 22 countries conducted the survey**
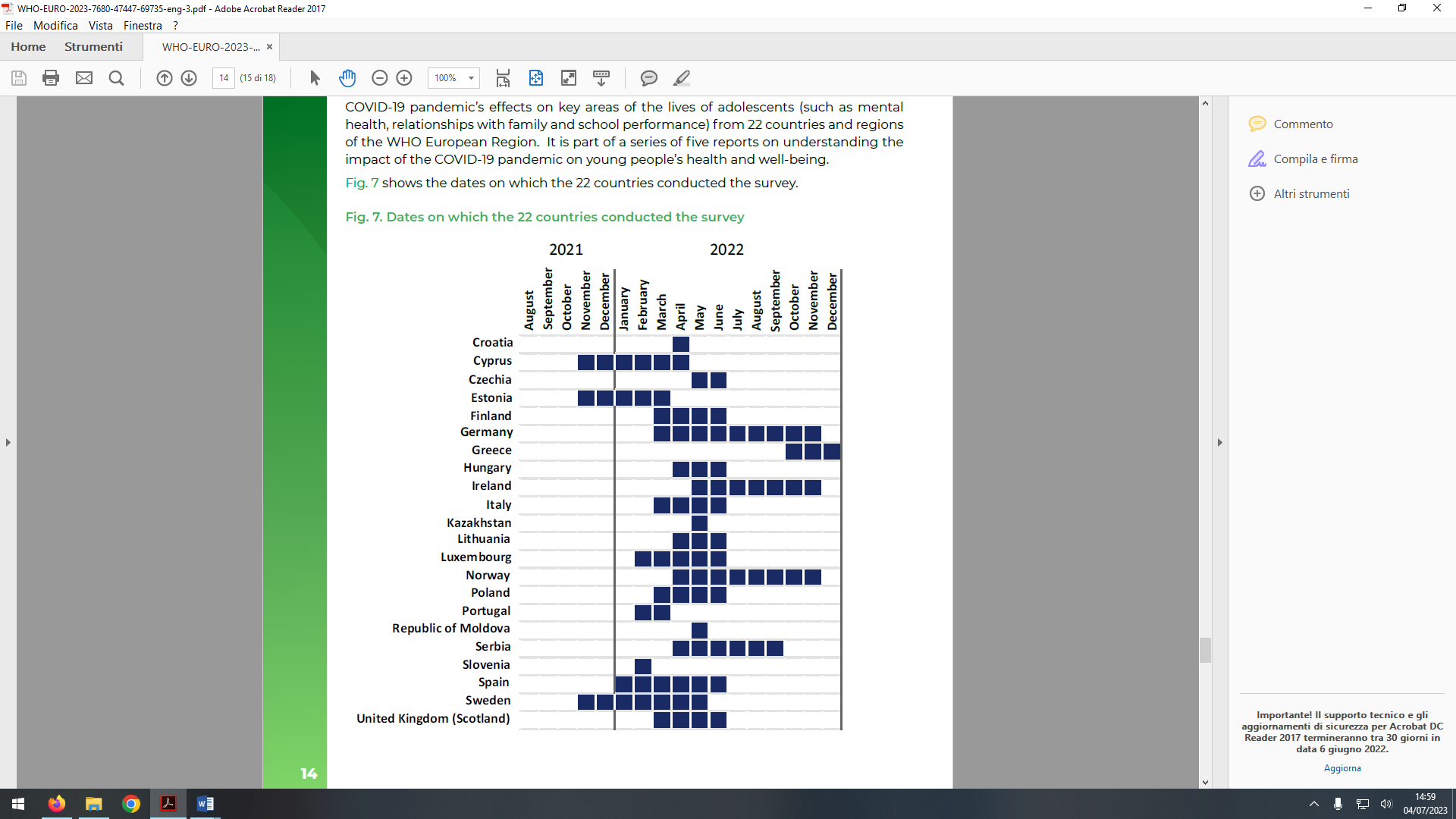


*Reprinted from Residori et al. (2023) with permission.*

**Table S1 HSBSC Questionnaire: Self-perceived impact of COVID-19 measures on various aspects of adolescent’s life, health and well-being**

| Since the start of the COVID-19 pandemic [please insert national terminology], the lives of many people have been affected (i.e., lockdowns, school closures, distance learning, and social distancing - please provide here national specific examples for the measures implemented to fight the virus during the COVID-19 pandemic).  What impact did these measures have on the following aspects of your life?  *(A negative impact means it made things worse, a positive impact means it made things better).*  *Please tick one box for each line.* | | | | | |
| --- | --- | --- | --- | --- | --- |
|  | Very negative | Quite negative | Neither positive nor negative | Quite positive | Very positive |
| Your life as a whole | ^1 ▢^ | ^2 ▢^ | ^3 ▢^ | ^4 ▢^ | ^5 ▢^ |
| Your health | ^1 ▢^ | ^2 ▢^ | ^3 ▢^ | ^4 ▢^ | ^5 ▢^ |
| Relationships with your family | ^1 ▢^ | ^2 ▢^ | ^3 ▢^ | ^4 ▢^ | ^5 ▢^ |
| Relationships with your friends | ^1 ▢^ | ^2 ▢^ | ^3 ▢^ | ^4 ▢^ | ^5 ▢^ |
| Your mental health (e.g., dealing with your emotions, stress etc) | ^1 ▢^ | ^2 ▢^ | ^3 ▢^ | ^4 ▢^ | ^5 ▢^ |
| Your school performance | ^1 ▢^ | ^2 ▢^ | ^3 ▢^ | ^4 ▢^ | ^5 ▢^ |
| Physical activity (e.g., sports, cycling, walks, etc) | ^1 ▢^ | ^2 ▢^ | ^3 ▢^ | ^4 ▢^ | ^5 ▢^ |
| What you ate or drank | ^1 ▢^ | ^2 ▢^ | ^3 ▢^ | ^4 ▢^ | ^5 ▢^ |
| Your future expectations (e.g. exams; jobs, etc.) | ^1 ▢^ | ^2 ▢^ | ^3 ▢^ | ^4 ▢^ | ^5 ▢^ |
| Your family financial situation | ^1 ▢^ | ^2 ▢^ | ^3 ▢^ | ^4 ▢^ | ^5 ▢^ |

**Table S2 Sample size per country, age and gender (N=67,544).**

|  | **Boys** | | **Girls** | |
| --- | --- | --- | --- | --- |
|  | **13 years** | **15 years** | **13 years** | **15 years** |
| **Croatia** | 865 | 701 | 874 | 819 |
| **Cyprus** | 716 | 620 | 763 | 663 |
| **Czechia** | 1697 | 1812 | 1638 | 1825 |
| **Estonia** | 782 | 761 | 781 | 706 |
| **Finland** | 447 | 353 | 493 | 454 |
| **Germany** | 872 | 843 | 927 | 981 |
| **Greece** | 749 | 971 | 868 | 1212 |
| **Hungary** | 483 | 685 | 462 | 901 |
| **Ireland** | 530 | 434 | 692 | 570 |
| **Italy** | 742 | 796 | 688 | 676 |
| **Kazakhstan** | 1062 | 1048 | 1148 | 1142 |
| **Lithuania** | 619 | 825 | 689 | 807 |
| **Luxembourg** | 601 | 628 | 627 | 644 |
| **Moldova** | 933 | 894 | 885 | 909 |
| **Norway** | 383 | 352 | 402 | 347 |
| **Poland** | 613 | 745 | 640 | 1041 |
| **Portugal** | 815 | 778 | 883 | 984 |
| **Scotland/ United Kingdom of Great Britain and Northern Ireland** | 738 | 551 | 728 | 508 |
| **Serbia** | 470 | 548 | 474 | 806 |
| **Slovenia** | 921 | 948 | 1009 | 1129 |
| **Spain** | 489 | 471 | 520 | 567 |
| **Sweden** | 599 | 554 | 665 | 553 |

**Table S3 Goodness of fit of the MLCA solutions (N=67,544)**

|  | **AIC** | **CAIC** | **BIC** | **entropy** | **Res.Df** |
| --- | --- | --- | --- | --- | --- |
| **2 class solution** | 1709219 | 1710252 | 1710150 | 0.92 | 67441 |
| **3 class solution** | 1616330 | 1617990 | 1617826 | 0.89 | 67379 |
| **4 class solution** | 1564721 | 1567008 | 1566782 | 0.88 | 67317 |
| **5 class solution** | 1530704 | 1533619 | 1533331 | 0.87 | 67255 |

**Table S4 Percentage d** **istribution of the COVID-19 measure impact items by COVID-19 measure impact class (N=67,544)**

| **Negative impact class** | | | | | | | | | | |
| --- | --- | --- | --- | --- | --- | --- | --- | --- | --- | --- |
|  | **life** | **health** | **family** | **friends** | **mental** | **school** | **physical activity** | **eating** | **future** | **finances** |
| **Very Negative** | 35.1 | 28.2 | 25.9 | 26.2 | 49.6 | 39.5 | 40.9 | 34.7 | 37.6 | 23.7 |
| **Quite negative** | 33.7 | 30.9 | 23.4 | 25.8 | 25.3 | 24.5 | 24.4 | 25.2 | 26.5 | 20.3 |
| **Neither negative or positive** | 22.4 | 27.7 | 27.9 | 24.0 | 14.0 | 18.7 | 17.3 | 23.9 | 23.1 | 37.7 |
| **Quite Positive** | 5.8 | 8.3 | 14.5 | 15.4 | 5.9 | 10.8 | 9.8 | 9.6 | 7.6 | 10.6 |
| **Very positive** | 3.0 | 5.0 | 8.4 | 8.5 | 5.2 | 6.4 | 7.7 | 6.6 | 5.3 | 7.7 |
|  |  |  |  |  |  |  |  |  |  |  |
| **Neutral impact class** | | | | | | | | | | |
|  | **life** | **health** | **family** | **friends** | **mental** | **school** | **physical activity** | **eating** | **future** | **finances** |
| **Very negative** | 2.9 | 1.3 | 1.0 | 1.6 | 6.0 | 4.8 | 5.3 | 2.6 | 2.0 | 1.5 |
| **Quite negative** | 19.1 | 13.8 | 7.8 | 13.4 | 23.6 | 23.6 | 25.3 | 15.9 | 14.4 | 10.3 |
| **Neither negative or positive** | 65.7 | 68.6 | 60.1 | 57.1 | 56.8 | 50.6 | 47.1 | 64.2 | 69.7 | 72.6 |
| **Quite positive** | 9.9 | 12.8 | 24.0 | 21.2 | 10.2 | 15.8 | 16.2 | 13.6 | 10.5 | 11.6 |
| **Very positive** | 2.4 | 3.5 | 7.1 | 6.7 | 3.5 | 5.2 | 6.0 | 3.8 | 3.4 | 4.0 |
|  |  |  |  |  |  |  |  |  |  |  |
| **Positive impact class** | | | | | | | | | | |
|  | **life** | **health** | **family** | **friends** | **mental** | **school** | **physical activity** | **eating** | **future** | **finances** |
| **Very Negative** | 3.3 | 1.6 | 1.3 | 1.4 | 4.3 | 3.1 | 3.5 | 2.3 | 2.4 | 1.5 |
| **Quite negative** | 7.4 | 4.8 | 3.3 | 4.2 | 9.0 | 9.0 | 8.5 | 5.2 | 5.5 | 3.7 |
| **Neither negative or positive** | 29.6 | 20.2 | 14.3 | 16.2 | 22.7 | 28.4 | 19.6 | 22.7 | 27.6 | 24.8 |
| **Quite Positive** | 37.3 | 41.1 | 35.0 | 37.8 | 33.8 | 35.5 | 31.6 | 37.7 | 36.3 | 36.6 |
| **Very positive** | 22.5 | 32.3 | 46.1 | 40.4 | 30.3 | 24.0 | 36.8 | 32.1 | 28.2 | 33.5 |

**Table S5 Percentage**  **distribution of the COVID-19 measure impact classes by country (N=67,544)**

|  | **COVID-19 measure impact class** | | |
| --- | --- | --- | --- |
|  | **Negative** | **Neutral** | **Positive** |
| **Cyprus** | 29,9 | 48,8 | 21,3 |
| **Czechia** | 17,8 | 44,2 | 38,1 |
| **Germany** | 12,1 | 56,6 | 31,3 |
| **Estonia** | 11,7 | 68,2 | 20,1 |
| **Spain** | 14,2 | 47,1 | 38,7 |
| **Finland** | 10,1 | 58,3 | 31,6 |
| **Scotland** | 8,5 | 69,7 | 21,7 |
| **Greece** | 26,8 | 59,6 | 13,6 |
| **Croatia** | 10,9 | 42,3 | 46,8 |
| **Hungary** | 26,4 | 60,6 | 13,0 |
| **Ireland** | 18,6 | 53,7 | 27,7 |
| **Italy** | 23,1 | 47,5 | 29,4 |
| **Kazakhstan** | 33,0 | 39,2 | 27,8 |
| **Lituania** | 20,0 | 71,6 | 8,4 |
| **Luxemburg** | 11,4 | 44,1 | 44,5 |
| **Moldova** | 8,3 | 40,2 | 51,5 |
| **Norway** | 9,6 | 44,1 | 46,4 |
| **Poland** | 26,7 | 61,4 | 11,9 |
| **Portugal** | 13,6 | 54,9 | 31,5 |
| **Serbia** | 18,1 | 52,8 | 29,1 |
| **Sweden** | 9,7 | 50,4 | 39,9 |
| **Slovenia** | 13,2 | 36,9 | 49,9 |

**Table S6 Percentage**  **gender, age, and SES distribution by COVID-19 measure impact class (N=67,544)**

|  | **Positive impact class** | **Neutral impact class** | **Negative impact class** | **p** |
| --- | --- | --- | --- | --- |
| **Gender** |  |  |  |  |
| Boys | 57.8 | 44.9 | 39.0 | <0.001 |
| Girls | 42.2 | 55.1 | 61.0 |  |
| **Age** |  |  |  |  |
| 13 yrs | 55.9 | 42.8 | 42.5 | <0.001 |
| 15 yrs | 44.1 | 57.2 | 57.5 |  |
| **SES** |  |  |  |  |
| Low | 22.1 | 24.9 | 31.1 | <0.001 |
| Medium | 62.5 | 63.6 | 58.4 |  |
| High | 15.4 | 11.5 | 10.5 |  |

*Abbreviations. SES, socioeconomic status; yrs, years.*

*Note. Chi-square tests, considering survey design effects (including stratification, clustering, and weighting) were performed.*

**Table S7 Percentage predicted probability of the explored mental outcomes by COVID-19 measure impact class among girls and boys (N=67,544)**

|  | | **Boys** | | | | | | | **Girls** | | | | |
| --- | --- | --- | --- | --- | --- | --- | --- | --- | --- | --- | --- | --- | --- |
|  | | **mean** | | | **95% CI** | | | | **mean** | | | **95% CI** | |
| **Loneliness** |  | |  | | | |  | | |  | | |  |
| Positive impact | | 5,8 | | | 4,5 - 7,2 | | | | 9,7 | | | 7,9 - 11,6 | |
| Neutral impact | | 9,8 | | | 8,2 - 11,3 | | | | 19,4 | | | 17,4 - 21,3 | |
| Negative impact | | 17,8 | | | 14,1 - 21,4 | | | | 44,3 | | | 40,0 - 48,6 | |
| **MHC (>=2 at least twice a week)** | |  | |  | |  | |  | | |  | |  |
| Positive impact | | 24 | | | 21,3 - 26,6 | | | | 49,7 | | | 46,1 - 53,4 | |
| Neutral impact | | 31,2 | | | 28,5 - 33,9 | | | | 62,7 | | | 60,3 - 65,1 | |
| Negative impact | | 45,1 | | | 40,0 - 50,1 | | | | 80,9 | | | 77,3 - 84,5 | |
| **High LS (>=9)** | |  | |  | |  | |  | | |  | |  |
| Positive impact | | 19,5 | | | 17,0 – 22,0 | | | | 19,1 | | | 16,4 - 21,7 | |
| Neutral impact | | 16,2 | | | 14,2 - 18,3 | | | | 12,5 | | | 10,8 - 14,1 | |
| Negative impact | | 11,2 | | | 8,1 - 14,3 | | | | 6,4 | | | 4,4 - 8,4 | |

*Note. Results adjusted by age and socioeconomic status.*

**Table S8 Association between the perceived COVID-19 measure impact and well-being, self-efficacy, and excellent self-rated health (N=67.544).**

|  | **Boys** | | | **Girls** | | |
| --- | --- | --- | --- | --- | --- | --- |
|  | **mean** | **2.5 %** | **97.5 %** | **mean** | **2.5 %** | **97.5 %** |
| **WHO-5 well-being (0-100 score) Beta** |  |  |  |  |  |  |
| **Positive impact (vs neutral)** | **5,70** | 3,73 | 7,67 | **7,14** | 5,13 | 9,14 |
| **Negative impact (vs neutral)** | -2,13 | -5,71 | 1,44 | **-6,10** | -8,39 | -3,81 |
| **Self-efficacy: doing things OR** |  |  |  |  |  |  |
| **Positive impact (vs neutral)** | **1,63** | 1,33 | 1,99 | **1,73** | 1,41 | 2,12 |
| **Negative impact (vs neutral)** | **0,71** | 0,53 | 0,94 | **0,57** | 0,46 | 0,72 |
| **Self-efficacy: finding solutions (OR)** |  |  |  |  |  |  |
| **Positive impact (vs neutral)** | 1,12 | 0,90 | 1,39 | **1,27** | 1,03 | 1,58 |
| **Negative impact (vs neutral)** | **0,56** | 0,42 | 0,75 | **0,56** | 0,45 | 0,70 |
| **Excellent self-rated health (OR)** |  |  |  |  |  |  |
| **Positive impact (vs neutral)** | **1,88** | 1,55 | 2,28 | **2,45** | 1,97 | 3,06 |
| **Negative impact (vs neutral)** | 1,24 | 0,94 | 1,65 | 0,80 | 0,61 | 1,06 |
